# The Value and Use of the Thermo-Cautery1Read before the Nebraska Veterinary Medical Association.

**Published:** 1898-08

**Authors:** George P. Tucker

**Affiliations:** Lincoln, Nebraska


### THE VALUE AND USE OF THE THERMO-CAUTERY.[1]

BY GEORGE P. TUCKER, V.S.,
LINCOLN, NEBRASKA.

THE thermo-cautery is one of the most valuable instruments that the veterinarian has, and while I do not wish to state that the hot iron is not a good instrument in the hands of an experienced operator, its great drawback is the impossibility of maintaining the proper temperature, and on this account should be discarded. The thermo-cautery overcomes this defect, besides being more easily handled, one hand being all that is required for its use. I speak of the improved form that carries the benzine in the handle.

[1] Read before the Nebraska Veterinary Medical Association.

Firing is the only radical cure for spavin, ringbone, sidebone, high splints, etc. It gives very good results in the treatment of chronic joint-lameness, and is of value in the treatment of muscular diseases. It has been used with gratifying results in the treatment of hogs suffering from partial loss of power in the hind parts, in which case good is effected by fairly deep firing along the spine and each side. Perhaps the best results from its use are seen in the treatment of sprains of ligaments and tendons. Many of our fast horses would be useless but for its curative effects. Probably no better example of its successful use can be given than in sprains or strains of the suspensory ligament in fast horses. That it works wonders in this case is beyond dispute.

There are various modes of procedure varying as to the ailment, but the best results are obtained by using the heated point boldly and as often over a given space as possible without causing a slonghing of the parts between the punctures.

I have spoken only of the pointed instrument, as I have failed to get any great benefit from the so-called line-firing, and I am of the opinion that it is of little if any use compared with the process of puncturing, which gives better results and does not blemish.

In firing it is seldom necessary to throw a horse, as the use of the twitch and the holding up of the opposite leg is generally sufficient.

In bone-diseases the puncture shoud be as deep as possible. In muscular affections firing through the skin is better than blistering alone. In such cases there is no special object in deep firing, except in the affection of pigs, mentioned above, when deep firing produces better results. In firing ligaments or back-tendons the production of scar-tissue is the main object, binding all three together, if possible. This also necessitates deep firing. In this, as well as in bone-firing, little or no attention need be paid to the puncturing of bursæ, as it is often impossible to bring about the desired results without so doing. In such cases, however, the wound should be kept aseptic.

After being fired the animal should be allowed to rest as long as possible, two months at least. In the treatment of the wound caused by the punctures little is to be done except to apply a blister within twenty-four hours after firing. In bone-diseases the best blister is red iodide of mercury (1 : 4). One application is all that is necessary, and need not be washed off. In tendon- and ligament-firing the best blister to be applied is a mixture of red iodide of mercury and cantharides (one-half of each to four). It will be found necessary to wash this off in a few days.

